# Infection of keratocystic odontogenic tumour by Pseudomonas aeruginosa

**DOI:** 10.1016/S1808-8694(15)30848-X

**Published:** 2015-10-18

**Authors:** Alvimar Lima de Castro, Evanice Menezes Marçal Vieira, Lívia Trevelin Arêde, Elerson Gaetti Jardim Junior

**Affiliations:** 1 Adjunct Professor of Stomatology - Dentistry School of Araçatuba/UNESP, Head of the Pathology and Clinical Propedeutics Department.; 2 PhD Student - Graduate Studies in Dentistry - Stomatology - Dentistry School of Unesp/Araçatuba, Professor - Dentistry School - Universidade de Cuiabá-UNIC/MT.; 3 Student - Faculdade de Odontologia de Araçatuba/UNESP; 4 Adjunct Professor of Microbiology and Immunology - Faculdade de Odontologia de Araçatuba/UNESP, Coordinator of the MCoordenador da disciplina de microbiologia da Faculdade de Odontologia de Araçatuba/UNESP. Faculdade de Odontologia de Araçatuba - UNESP

**Keywords:** bone cysts, focal infection, pseudomonas, drug resistance

## INTRODUCTION

The oral cavity may represent a reservoir of Pseudomonas aeruginosa, especially in patients with periodontitis; this makes treatment more difficult and, in the case of opportunistic infections, may worsen the conditions of debilitated patients, such as the elderly and immunossupressed^1^, ^2^. The present investigation aims at using a clinical case to discuss the possibility of a secondary infection of a keratocystic odontogenic tumor by P. aeruginosa multiresistant to antimicrobial agents.

## CASE REPORT

**Figure 1:**
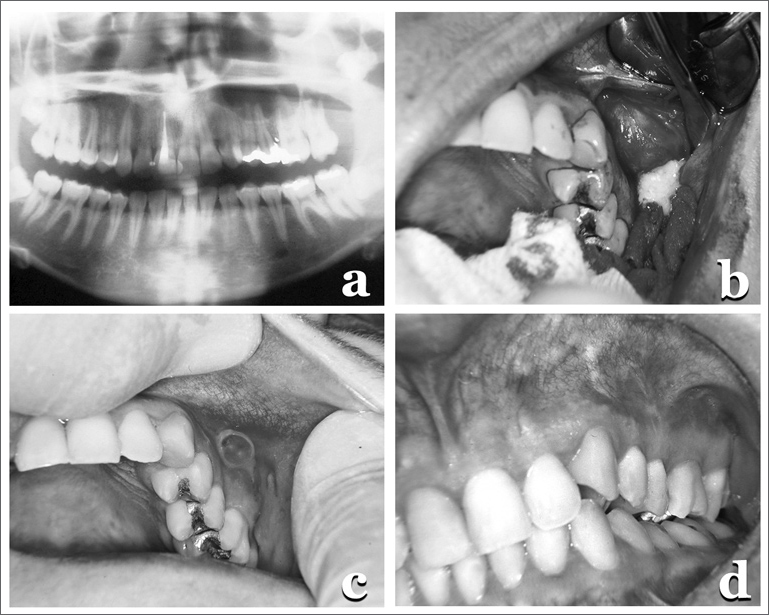
(a) Panoramic X-ray showing a radiolucent, unilocular area, well outlined, in the region of the canine to the first molar in the right side, involving the unerupted tooth; (b) Surgical cavity with the cyst, the ruptured capsule, showing a whitish keratin cluster; (c) fistula; (d) Final clinical case with a normal mucosa.

A 24 year-old female patient came with a panoramic radiography that showed an unerupted upper third molar associated with a radiolucent lesion (Fig. 1a), later on diagnosed as keratocystic odontogenic tumor (Fig. 1b).

Two weeks after lesion enucleation, a fistula appeared (Fig. 1c), with a buccosinusal communication and oozing of a yellowish fluid that was aspirated and sent for culture - carried out in blood agar in aerobiosis and anaerobiosis, at 37°C, for 48 hours and 15 days, respectively. The isolated micro-organisms were identified by means of biochemical tests, showing only the presence of Pseudomonas aeruginosa.

The microorganism was submitted to antimicrobial susceptibility tests: amikacin, amoxicillin, amoxicillin/clavulanic acid, azithromycin, chloramphenicol, ciprofloxacin, clindamycin, doxicycline, erythromycin, spiramycin, streptomycin, imipenem, lincomycin, norfloxacin, G penicillin, rifampin, tetracycline, tobramycin, vancomycin, and it was resistant to all of them, with the resistance varying from 64mg/ml to 512 mg/ml, much above what could be clinically reached. The bacteria produced b-lactamase (s) capable of degrading all the b-lactamic drugs.

In the 72 hour period between clinical specimen collection and the antibiogram results, the patient was medicated with amoxicillin without satisfactory results. We then decided to terminate the use of antibiotics and we performed the surgery. The fistula remained.

In search of the factors associated with keeping the infection, by means of a radiographic analysis we identified pulpar involvement in the second upper left molar, which received endodontic treatment. Through the fistula, we removed organic remains from there using water/hydrogen peroxide (equal volumes) with satisfactory results (Fig. 1d).

## DISCUSSION

Pseudomonas in the oral cavity is not a rare occurrence; however, its multiple resistance and the involvement of root canals is not common, since endodontic infections are usually associated to a mixed microbiota with a predominance of anaerobic bacteria, and Pseudomonas aeruginosa is aerobic^3^.

Nonetheless, in asymptomatic endodontic infections the microbiota proved to be mainly made up of facultative anaerobic and aerobic bacteria^4^, in such a way that the origin of this secondary infection in the keratocystic tumor is possibly associated with the dental disease. However, since we did not see any communication between the root canal system and the external environment or the presence of periodontitis, we can not safely state how the microorganism reached pulpar and periapical tissue. Nonetheless, the surgical trauma, despite all care taken with asepsis, allowed for its spread towards the tissues of the oral cavity and those of the maxillary sinus.

We must take exceptional care with the systemic spread of these multiresistant rods, and we submit that the major factor responsible for the successful treatment was the favorable health condition of the patient and the proper endodontic treatment that was undertaken. Bacteria of the Pseudomonas genus frequently have multiple resistance mechanisms, and in the case of b-lactamic, it happened because of the production of b-lactamases and, it is likely also associated with the development of water tight barriers^5^, ^6^.

## FINAL REMARKS

Pseudomonas infections of the oral cavity is not common, and the microbiologic test must be always carried out when one suspects of such infection, since because of its multiresistance, there is no drug treatment widely acknowledged as efficient. By observing the traits present in this report, we must stress that the clinician must be attentive to the possibilities of infection with uncommon characteristics in the oral cavity and the spread of such infections caused by surgical procedures.
